# Introducing a Standardized Clinical Investigation Sheet for Medical Documentation at Dongola Specialized Hospital, Sudan: A Quality Improvement Project

**DOI:** 10.7759/cureus.92512

**Published:** 2025-09-17

**Authors:** Abubakr Muhammed, Mohaned Altijani Abdalgadir Hamdnaalla, Fakher Aldeen Raft Fakher Aldeen Noman, Mohammed Ali Mohammed Ali, Mohey Aldien Ahmed Elamin Elnour, Awad Elkarim Adil Awad Elkarim Mohamed, Suzan Mohammed Eltayeb Eltahir, Rawa Mohamed Idris Mohamed, Ahmed Shakir Ali Yousif, Bashaier Idris Mohamed Abdelrahman, Maysara Elsiddig, Ibrahim Adil Hamadelniel Alhadi, Thoiba Mohammed Hamdnaallah Mohammed, Heif Aljenan Mohammed Ahmed Yousif Mohammed, Thwiaba Abdelgadir Yousif Mustafa, Mohammed Osman Ahmed Osman, Fatima Awad Khalil Mohammed, Areej Osman Adam Osman, Musaab Ahmed Ali Fadul, Abdullah Mohamed

**Affiliations:** 1 Surgery, University of Gezira, Madani, SDN; 2 Internal Medicine, Dongola Specialized Hospital, Dongola, SDN; 3 Neurosurgery, University Hospitals Coventry and Warwickshire NHS Trust, Coventry, GBR; 4 General Surgery, Port Sudan Teaching Hospital, Port Sudan, SDN; 5 Internal Medicine, Sudan Medical Specialization Board, Khartoum, SDN; 6 Paediatrics, Dongola Specialized Hospital, Dongola, SDN; 7 Urology, Dongola Specialized Hospital, Dongola, SDN; 8 General Surgery, Burjeel Medical City, Abu Dhabi, ARE; 9 General Surgery, Dongola Specialized Hospital, Dongola, SDN; 10 Pediatric Medicine, Sudan Medical Specialization Board, Madani, SDN; 11 Urology, University Hospitals of Morecambe Bay, Royal Lancaster Infirmary, Lancaster, GBR; 12 Internal Medicine, National Ribat University, Khartoum, SDN; 13 General Practice, Dongola Specialized Hospital, Dongola, SDN

**Keywords:** clinical audit cycles, healthcare compliance rates, medical documentation gaps, patient safety interventions, quality improvement project, resource-limited settings, standardized investigation sheet, structured documentation tools, sudan healthcare improvement

## Abstract

Inconsistent and incomplete documentation of investigation results at Dongola Specialized Hospital in Sudan has compromised patient safety and clinical efficiency. To address this, a two-cycle quality improvement project was conducted between April and May 2025, with targeted interventions implemented between the cycles to enhance compliance and address identified gaps. In the first cycle, root causes such as the absence of standardized forms, inadequate staff training, and a lack of accountability mechanisms were identified. Interventions included the introduction of a standardized investigation sheet, targeted staff education, and reinforcement strategies such as instructional posters and laminated samples. In the second cycle, redesigned forms with mandatory fields and visual cues were implemented, resulting in substantial improvements in documentation. Patient identification improved from 1.9% to 76.9%, critical markers such as blood group, hepatitis, and HIV status were consistently recorded (100%), and overall documentation completeness rose from 18.2% to 74.0%. However, persistent gaps remained in time-sensitive investigations such as arterial blood gases and glucose levels. These findings demonstrate that standardized documentation tools, supported by training and accountability, can significantly strengthen medical record quality and, by extension, enhance patient safety and clinical decision-making in resource-limited settings. Sustained monitoring, targeted refinements, and potential integration with electronic health records are recommended to maintain progress and scale improvements across similar contexts.

## Introduction

Medical documentation is a cornerstone of safe and effective healthcare delivery, serving as the foundation for clinical decision-making, continuity of care, and medico-legal accountability [1]. Inadequate or inconsistent documentation can compromise patient safety, fragment care, and lead to adverse outcomes, prolonged hospital stays, and higher costs [1]. Deficient documentation has also been linked to delayed interventions, adverse events, and increased healthcare costs, particularly in resource-limited systems [2].

In sub-Saharan Africa, including Sudan, persistent challenges with documentation quality have been documented. Studies from Uganda and Ethiopia highlight incomplete, illegible, and poorly integrated records that increase the risk of errors and limit care coordination [2]. Audits at regional referral hospitals have also shown that both doctors and nurses frequently fail to document essential aspects of care, reducing interprofessional collaboration and visibility of the clinical process [2-5].

Quality improvement (QI) initiatives have demonstrated the effectiveness of structured documentation tools, such as standardized checklists and investigation sheets, in improving record completeness and accuracy [2-8]. These approaches align with World Health Organization (WHO) recommendations, which emphasize standardized tools, continuous monitoring, and the potential of electronic health records (EHRs) to enhance quality and safety [6-14].

Building on this evidence, the present project contributes novel data from Sudan through a two-cycle audit at Dongola Specialized Hospital. This project aimed to design and implement a standardized investigation sheet to improve the completeness and accuracy of medical documentation at Dongola Specialized Hospital. By aligning with the “triple aim” framework - improving care, health, and cost - this work provides a replicable model for resource-limited settings while addressing a critical gap in the local literature [3-11].

## Materials and methods

Study design and setting

This prospective quality improvement project was conducted at Dongola Specialized Hospital between April 1 and May 15, 2025, with the aim of evaluating the completeness and quality of medical investigation documentation. A two-cycle audit design was employed, in line with best practices in quality improvement, to allow for baseline assessment, targeted intervention, and re-evaluation.

First Cycle: Pre-intervention Assessment and Root Cause Analysis (April 1-15, 2025)

**Figure 1:**
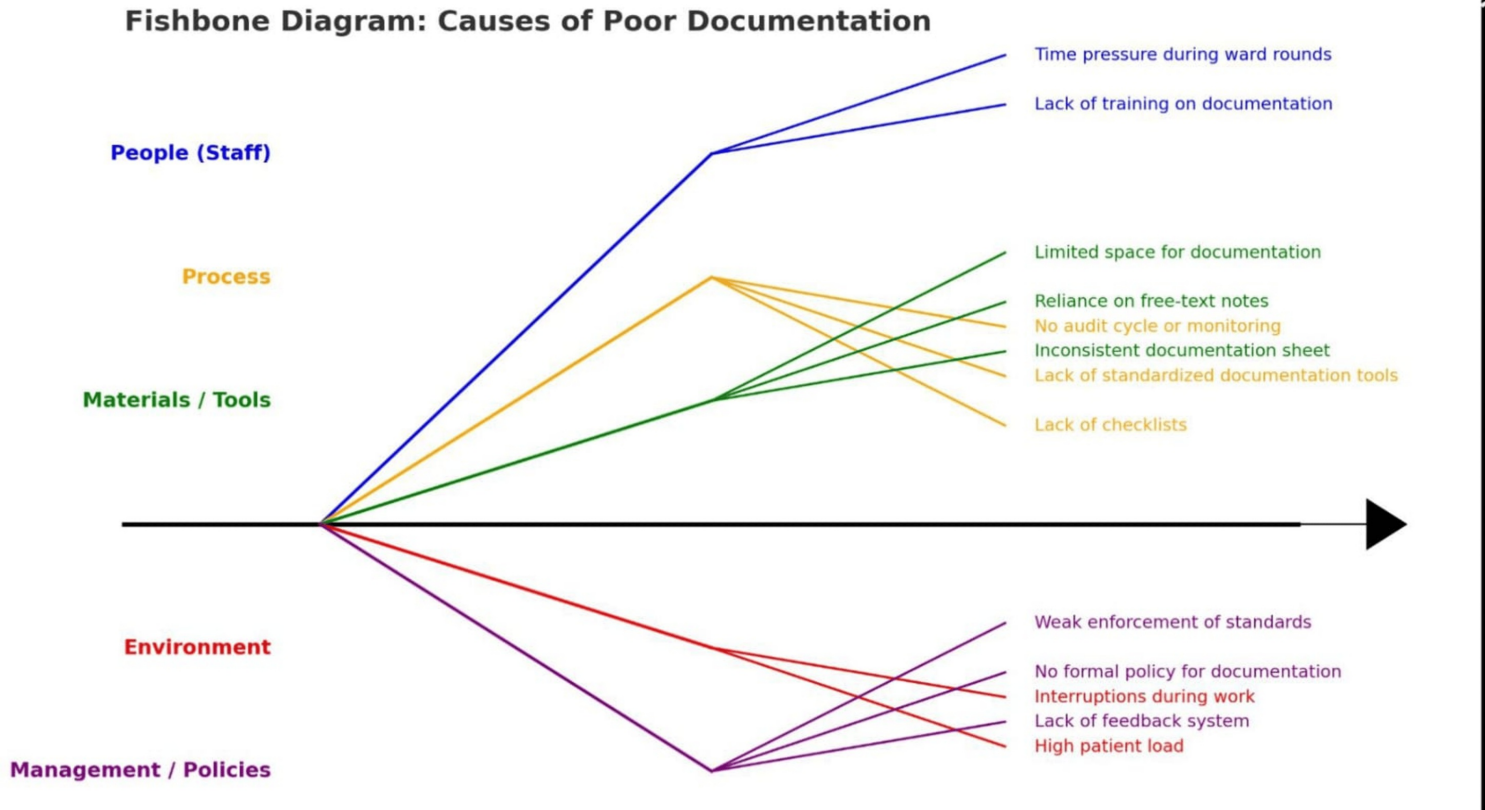
Causes of Poor Documentations

To address this, a two-cycle audit was conducted between April and May 2025, with targeted interventions implemented between the cycles to enhance compliance and address identified gaps. As part of the baseline assessment, a prospective evaluation of patient investigation sheets was conducted, reviewing 53 randomly selected forms from the General Surgery Unit to assess documentation quality prior to intervention. These forms included laboratory results generated during routine inpatient care and were analyzed for completeness, adherence to protocol, and clarity of clinical justification. Selection was made through simple random sampling from all available forms during the audit period, thereby minimizing selection bias. Data collection included chart audits and structured staff interviews. A root cause analysis was undertaken using multidisciplinary focus group discussions with physicians, nurses, and laboratory personnel. Fishbone diagrams were employed to identify barriers, highlighting the absence of standardized forms, inadequate training, and a lack of accountability mechanisms (Figure 1).

Intervention: Standardization and Training (April 16-30, 2025)

Based on the identified gaps, a standardized investigation sheet was developed (Appendix 1), incorporating mandatory fields such as patient and doctor identifiers, test names, collection dates and times, sequential result entries, and staff signatures. The design adhered to WHO documentation standards and included visual cues to reinforce completion.

To ensure effective implementation, two half-day training workshops were conducted, attended by 41 staff members (physicians, nurses, and laboratory personnel). Training sessions included short lectures on the medico-legal and clinical importance of documentation, guided demonstrations of the new form, and practical exercises in form completion. Reinforcement strategies included distribution of laminated sample forms to departments, display of instructional posters in clinical units, and follow-up coaching by designated documentation champions. Attendance records were maintained, though no formal competency assessments were performed.

Second Cycle: Post-intervention Audit and Feedback (May 1-15, 2025)

A second audit reviewed 52 randomly selected forms from the intervention period, again using simple random sampling to ensure comparability with baseline data.

Data analysis

The quality of documentation was assessed by calculating completion percentages for 17 defined domains. Pre- and post-intervention results were compared using chi-square tests for categorical variables, with statistical significance set at p < 0.05. Results were then summarized to identify domains showing significant improvement, as well as persistent gaps requiring further intervention.

Ethical approval

Ethical approval was granted by the Institutional Review Board at Dongola Specialized Hospital (approval number IRB 2025-QIP-000975). All data were anonymized to protect patient confidentiality throughout collection and analysis.

## Results

In the first cycle (pre-intervention, N = 53), documentation of investigation results at our centre was markedly deficient across most assessed domains. Patient identification was recorded in only 1.9% of forms, while critical infection markers such as hepatitis, HIV, and blood group status were documented in fewer than 4% of cases. Consistent date and time entries were missing in more than 90% of forms. Documentation of sequential laboratory investigations, including complete blood counts and renal function tests, was inconsistent, and time-sensitive investigations such as arterial blood gases (ABG) and glucose levels were rarely documented. The overall mean completion percentage across all domains was 18.2%.

Following the introduction of the standardized investigation sheet and staff training, documentation quality improved substantially in the second cycle (post-intervention, N = 52). Patient identification increased to 76.9% (p < 0.001), and documentation of critical infection markers (blood group, hepatitis, HIV status) reached 100% (p < 0.001). Sequential recording of laboratory results improved across multiple domains, including CBC (92.3% vs 49.1%, p < 0.001) and renal function (92.3% vs 52.8%, p < 0.001). Time-sensitive parameters such as ABG and glucose improved to 61.5% and 73.1%, respectively, though these remained below optimal compliance levels. Overall documentation completeness increased from 18.2% at baseline to 74.0% after intervention (p < 0.001).

These results, summarized in Table 1, demonstrate significant improvements across most parameters.

**Table 1:** Documentation Completeness Before and After Intervention CDC: complete blood count; ABG: arterial blood gas

Category	Parameter	First Cycle; N=53 (%)	Second Cycle; N=52 (%)	p-value
Patient identifiers	Patient name	1 (1.9%)	40 (76.9%)	<0.001
	Doctor’s name	0 (0.0%)	48 (92.3%)	<0.001
Infection markers	Blood group	1 (1.9%)	52 (100%)	<0.001
	Hepatitis B status	2 (3.8%)	52 (100%)	<0.001
	Hepatitis C status	2 (3.8%)	52 (100%)	<0.001
	HIV status	2 (3.8%)	52 (100%)	<0.001
Hematology and inflammatory markers	CBC sequential with dates	26 (49.1%)	48 (92.3%)	<0.001
	Inflammatory markers sequential with dates	15 (28.3%)	45 (86.5%)	<0.001
Other lab investigations	Malaria test with date	16 (30.2%)	38 (73.1%)	<0.001
	Renal function sequential with dates	28 (52.8%)	48 (92.3%)	<0.001
	Serum calcium sequential with dates	7 (13.2%)	30 (57.7%)	<0.001
	Liver function sequential with dates	8 (15.1%)	33 (63.5%)	<0.001
	Uric acid with date	1 (1.9%)	29 (55.8%)	<0.001
	Urine analysis sequential with dates	13 (24.5%)	37 (71.2%)	<0.001
	Bleeding profile sequential with dates	2 (3.8%)	28 (53.8%)	<0.001
Time-sensitive investigations	ABG levels with time/date	8 (15.1%)	32 (61.5%)	<0.001
	Blood sugar with time/date	20 (37.7%)	38 (73.1%)	<0.001

## Discussion

Medical documentation is central to patient safety, continuity of care, and medico-legal accountability [1]. In many low- and middle-income countries, including Sudan, deficiencies in record-keeping compromise care quality and increase the risk of adverse outcomes [2,5].

This project demonstrated that introducing a standardized investigation sheet, combined with structured training and reinforcement strategies, markedly improved documentation quality at Dongola Specialized Hospital. The findings are consistent with reports from other sub-Saharan African hospitals, such as Wallaga University Referral Hospital in Ethiopia [2], and with Sudanese audits showing that standardized templates improved discharge documentation [5,8]. Compared with these benchmarks, the present study highlights the importance of coupling standardized tools with accountability mechanisms and reinforcement measures such as coaching and feedback to foster sustainable improvements [6,7,12].

Beyond record completeness, the intervention fostered a culture of accountability and consistency. Reinforcement measures, including mandatory form checks, laminated job aids, and feedback sessions, align with WHO recommendations that emphasize standardization, monitoring, and practical tools to strengthen health service quality [6,13,14]. These low-cost, context-appropriate strategies may be replicated in other resource-limited hospitals.

Despite significant progress, gaps persisted in documenting time-sensitive investigations such as arterial blood gases and glucose levels. Likely contributing factors include workflow pressures, the urgency of clinical care in critical situations, and form design limitations for high-frequency testing. Similar barriers have been reported in other settings, where incomplete records are linked to delays in interventions and potential safety risks [3,15]. Addressing these challenges may require simplified formats for urgent tests, visual “critical value” cues, or eventual integration with electronic health records [6,13,14].

This study has several strengths. The use of a two-cycle audit allowed for baseline assessment and re-evaluation, consistent with best practice in quality improvement. Root cause analysis using multidisciplinary focus groups ensured that the intervention was informed by local barriers and stakeholder perspectives. The standardized sheet was designed according to WHO recommendations, and reinforcement strategies such as staff training, coaching, and mandatory rejection of incomplete forms enhanced compliance and sustainability. These design elements strengthen the applicability of the findings to other resource-limited contexts.

However, the study also has important limitations. The sample size was modest, and the follow-up period was short, limiting assessment of sustainability over time. As a single-site project, generalizability is restricted. Although chi-square testing confirmed significant improvements across most domains, more advanced statistical analyses could provide greater robustness. Finally, formal competency assessments following training were not conducted, limiting conclusions regarding individual-level performance.

Despite these limitations, this project adds valuable evidence from Sudan, where systematic evaluations of documentation improvement remain scarce. By aligning with WHO recommendations and the “triple aim” of improving care, health, and cost [3,10,11], this initiative offers a practical and replicable model for strengthening clinical documentation in resource-limited contexts.

## Conclusions

The introduction and iterative refinement of a standardized investigation sheet at Dongola Specialized Hospital led to substantial and sustained improvements in documentation quality, addressing critical gaps and enhancing patient safety. Nevertheless, the persisting deficiencies in documenting time-sensitive investigations underscore the need for targeted strategies such as urgent-case flags, simplified formats for high-volume tests, and future integration with electronic health records to ensure timely recording of parameters critical to acute patient care.

## Figures and Tables

**Figure 2:**
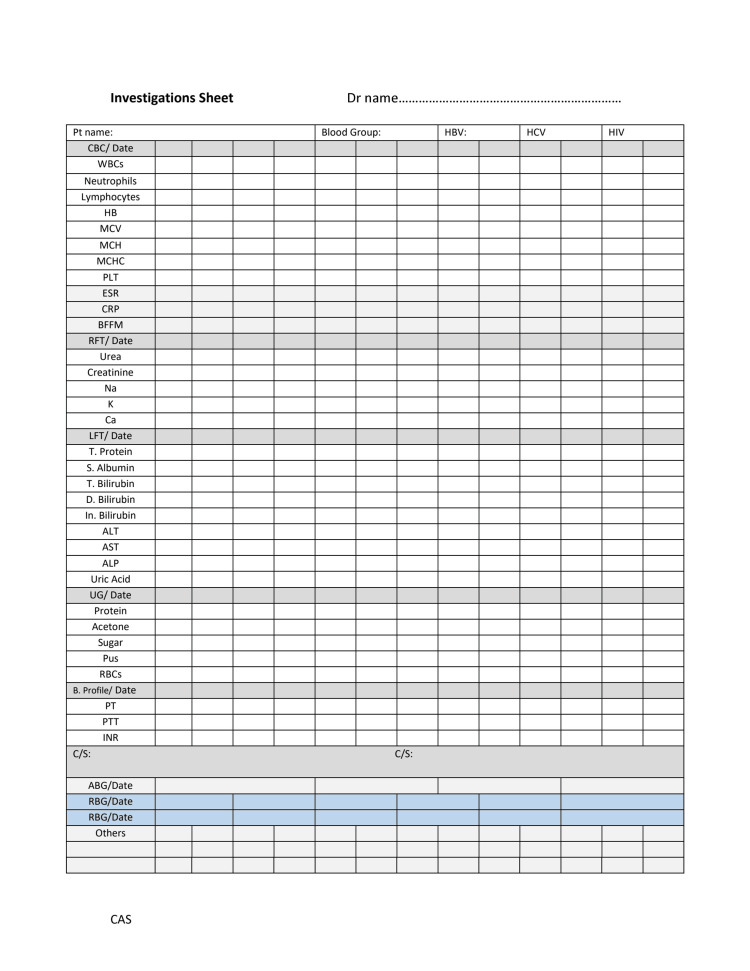
The New Investigation Sheet

## Appendices

Appendix 1

Based on the identified gaps, a standardized investigation sheet was developed (Figure 2). 

Appendix 2

Table 2 shows the questionnaire distributed to assess the documentation of clinical investigations before and after the intervention. 

**Table 2:** Clinical Investigation Documentation Questionnaire

Section	Question	Response Options
Patient Identification	Is the patient's name written clearly?	Yes / No
	Page number	[Open field]
	Is the doctor's name written clearly?	Yes / No
Basic Information	Is the patient's blood group written clearly?	Yes / No
	Is the patient's hepatitis B status written clearly?	Yes / No
	Is the patient's hepatitis C status written clearly?	Yes / No
	Is the patient's HIV status written clearly?	Yes / No
Investigations	Are CBC investigations written clearly in sequence with dates?	Yes / No
	Are inflammatory markers written clearly in sequence with dates?	Yes / No
	Is the malaria test clearly written with specific date?	Yes / No
	Are renal function results written clearly in sequence with dates?	Yes / No
	Is serum calcium level written clearly in sequence with dates?	Yes / No
	Are liver function test results written clearly in sequence with dates?	Yes / No
	Is uric acid level clearly written with specific date?	Yes / No
	Is urine analysis written clearly in sequence with dates?	Yes / No
	Is bleeding profile analysis written clearly in sequence with dates?	Yes / No
	Are ABG levels written clearly in sequence with specific time and dates?	Yes / No
	Are blood sugar levels written clearly in sequence with specific time and dates?	Yes / No
